# Supplementary material for: Genome Evolution and Introgression in the New Zealand mud Snails Potamopyrgus estuarinus and Potamopyrgus kaitunuparaoa
Source: Genome Biol Evol. 2024 May 22;16(5):evae091. doi: 10.1093/gbe/evae091 (PMC11110935; doi:10.1093/gbe/evae091)
Supplement: evae091_Supplementary_Data [file evae091_supplementary_data.zip › Suppl_Figures.pdf]

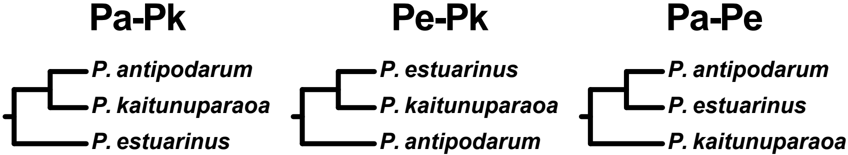
Pa-Pk
P. antipodarum
P. kaitunuparaoa
P. estuarinus
Pe-Pk
P. estuarinus
P. kaitunuparaoa
P. antipodarum
Pa-Pe
P. antipodarum
P. estuarinus
P. kaitunuparaoa

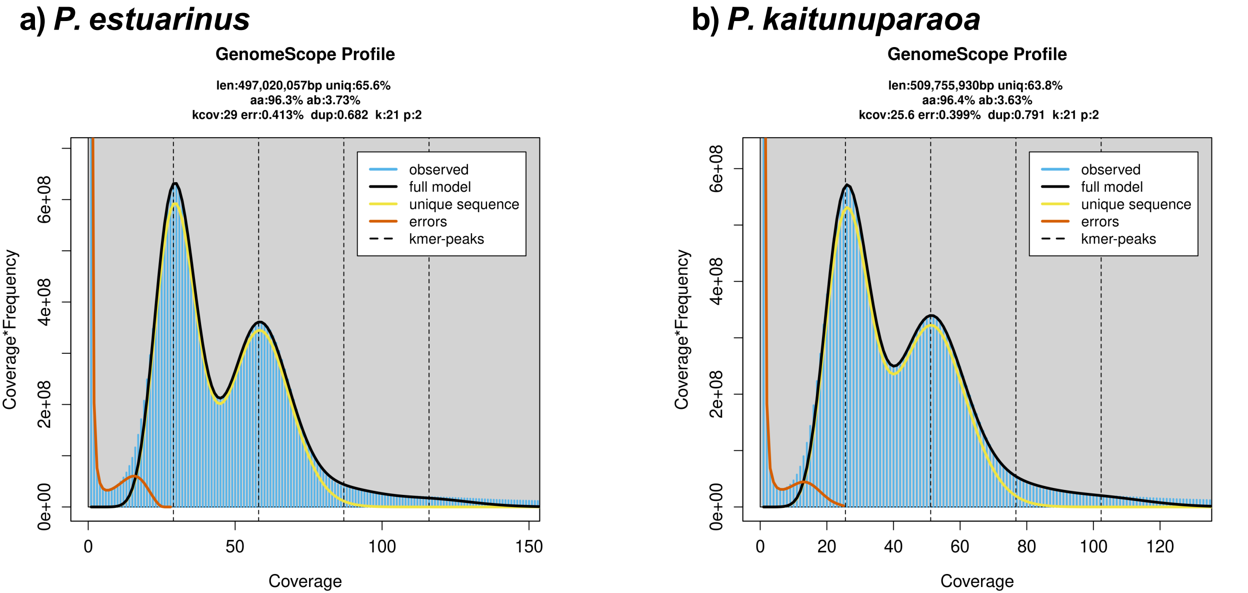
a) P. estuarinus
GenomeScope Profile
len:497,020,057bp uniq:65.6%
aa:96.3% ab:3.73%
kcov:29 err:0.413% dup:0.682 k:21 p:2
observed
full model
unique sequence
errors
kmer-peaks
Coverage*Frequency
0e+00
2e+08
4e+08
6e+08
0
50
100
150
Coverage
b) P. kaitunuparaoa
GenomeScope Profile
len:509,755,930bp uniq:63.8%
aa:96.4% ab:3.63%
kcov:25.6 err:0.399% dup:0.791 k:21 p:2
observed
full model
unique sequence
errors
kmer-peaks
Coverage*Frequency
0e+00
2e+08
4e+08
6e+08
0
20
40
60
80
100
120
Coverage

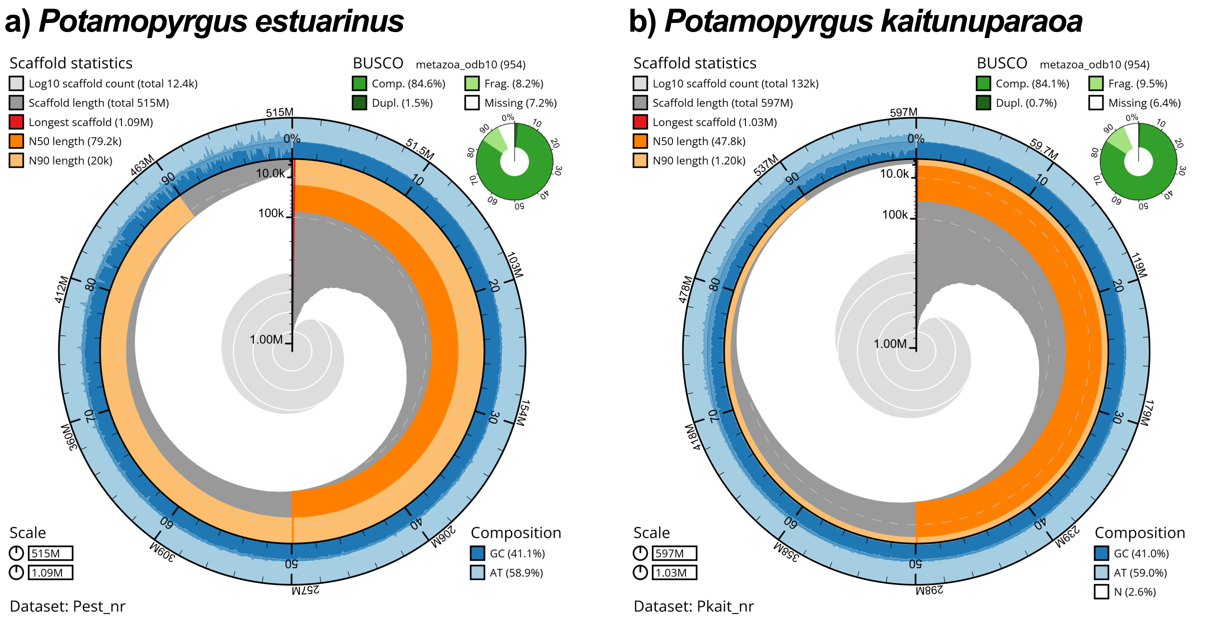
a) Potamopyrgus estuarinus
Scaffold statistics
Log10 scaffold count (total 12.4k)
Scaffold length (total 515M)
Longest scaffold (1.09M)
N50 length (79.2k)
N90 length (20k)
BUSCO metazoa_odb10 (954)
Comp. (84.6%)
Frag. (8.2%)
Dupl. (1.5%)
Missing (7.2%)
Scale
515M
1.09M
Composition
GC (41.1%)
AT (58.9%)
Dataset: Pest_nr
b) Potamopyrgus kaitunuparaoa
Scaffold statistics
Log10 scaffold count (total 132k)
Scaffold length (total 597M)
Longest scaffold (1.03M)
N50 length (47.8k)
N90 length (1.20k)
BUSCO metazoa_odb10 (954)
Comp. (84.1%)
Frag. (9.5%)
Dupl. (0.7%)
Missing (6.4%)
Scale
597M
1.03M
Composition
GC (41.0%)
AT (59.0%)
N (2.6%)
Dataset: Pkait_nr

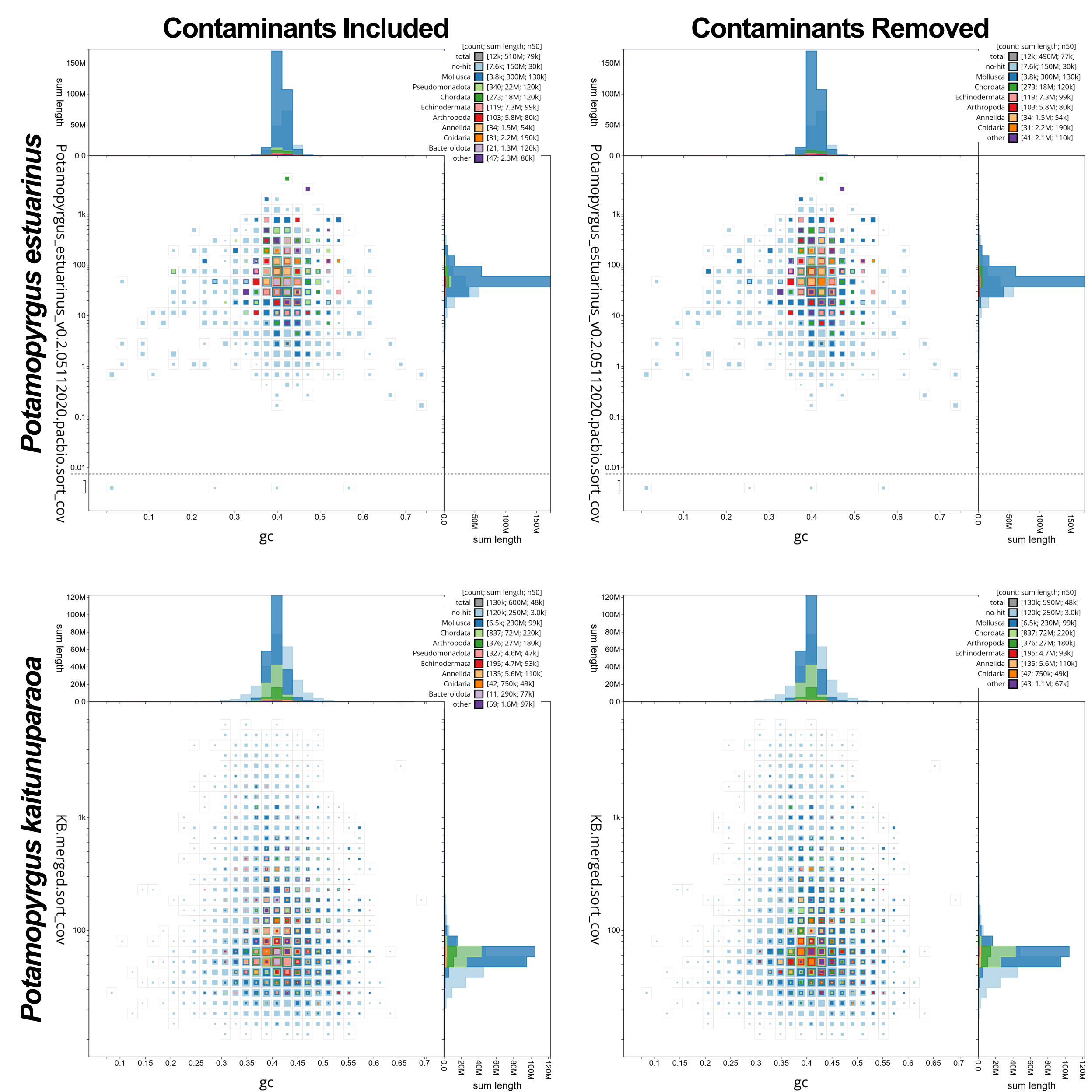
Contaminants Included
Contaminants Removed
Potamopyrgus estuarinus
Potamopyrgus kaitunuparaoa
Potamopyrgus_estuarinus_v0.2.05112020.pacbio.sort_cov
KB.merged.sort_cov
[count; sum length; n50]
total [12k; 510M; 79k]
no-hit [7.6k; 150M; 30k]
Mollusca [3.8k; 300M; 130k]
Pseudomonadota [340; 22M; 120k]
Chordata [273; 18M; 120k]
Echinodermata [119; 7.3M; 99k]
Arthropoda [103; 5.8M; 80k]
Annelida [34; 1.5M; 54k]
Cnidaria [31; 2.2M; 190k]
Bacteroidota [21; 1.3M; 120k]
other [47; 2.3M; 86k]
[count; sum length; n50]
total [12k; 490M; 77k]
no-hit [7.6k; 150M; 30k]
Mollusca [3.8k; 300M; 130k]
Chordata [273; 18M; 120k]
Echinodermata [119; 7.3M; 99k]
Arthropoda [103; 5.8M; 80k]
Annelida [34; 1.5M; 54k]
Cnidaria [31; 2.2M; 190k]
other [41; 2.1M; 110k]
[count; sum length; n50]
total [130k; 600M; 48k]
no-hit [120k; 250M; 3.0k]
Mollusca [6.5k; 230M; 99k]
Chordata [837; 72M; 220k]
Arthropoda [376; 27M; 180k]
Pseudomonadota [327; 4.6M; 47k]
Echinodermata [195; 4.7M; 93k]
Annelida [135; 5.6M; 110k]
Cnidaria [42; 750k; 49k]
Bacteroidota [11; 290k; 77k]
other [59; 1.6M; 97k]
[count; sum length; n50]
total [130k; 590M; 48k]
no-hit [120k; 250M; 3.0k]
Mollusca [6.5k; 230M; 99k]
Chordata [837; 72M; 220k]
Arthropoda [376; 27M; 180k]
Echinodermata [195; 4.7M; 93k]
Annelida [135; 5.6M; 110k]
Cnidaria [42; 750k; 49k]
other [43; 1.1M; 67k]
sum length
gc

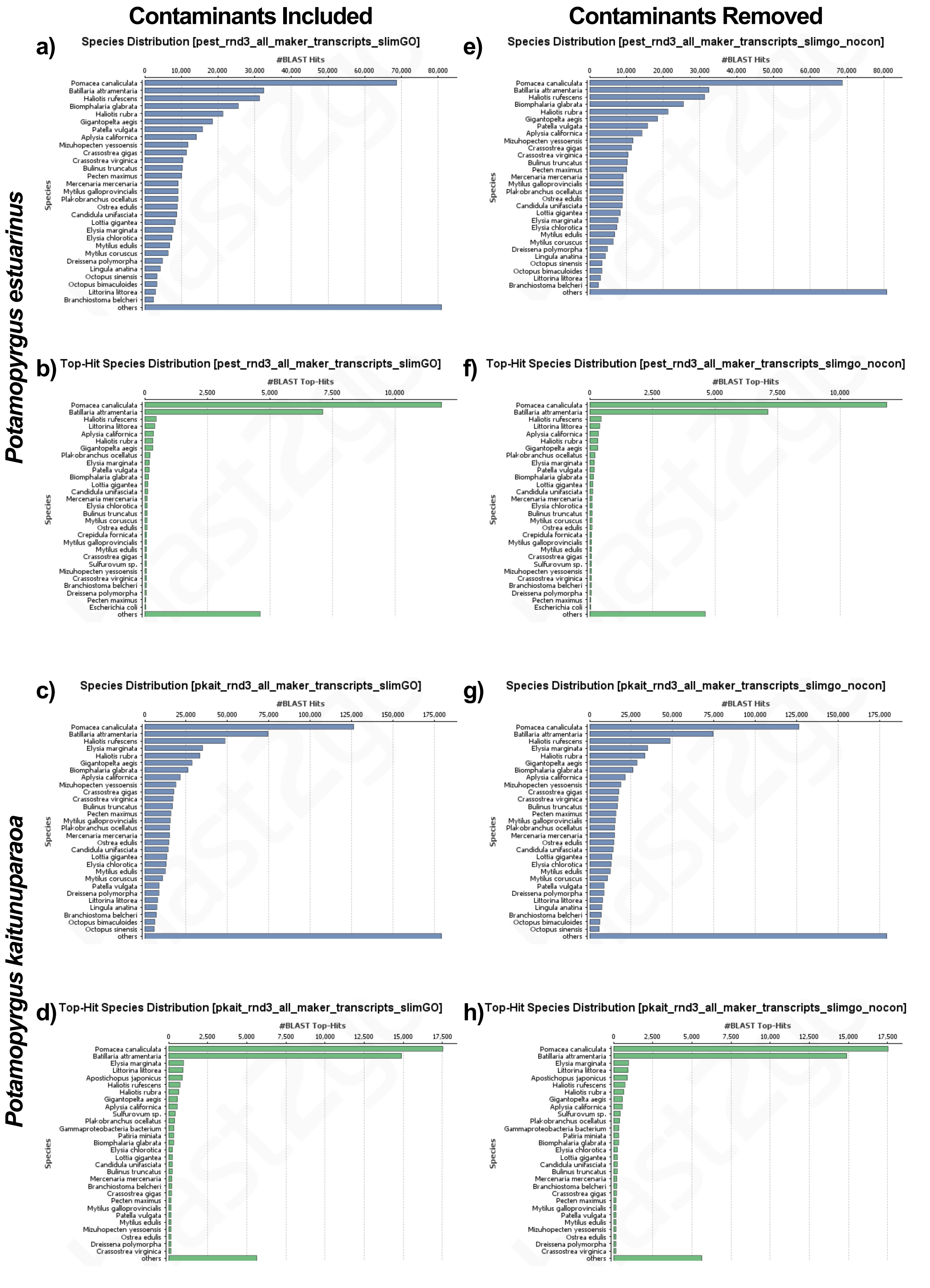

Contaminants Included
Contaminants Removed
Potamopyrgus estuarinus
Potamopyrgus kaitunuparaoa
a)
Species Distribution [pest_rnd3_all_maker_transcripts_slimGO]
#BLAST Hits
0
10,000
20,000
30,000
40,000
50,000
60,000
70,000
80,000
Species
Pomacea canaliculata
Batillaria attramentaria
Haliotis rufescens
Biomphalaria glabrata
Haliotis rubra
Gigantopelta aegis
Patella vulgata
Aplysia californica
Mizuhopecten yessoensis
Crassostrea gigas
Crassostrea virginica
Bulinus truncatus
Pecten maximus
Mercenaria mercenaria
Mytilus galloprovincialis
Plakobranchus ocellatus
Ostrea edulis
Candidula unifasciata
Lottia gigantea
Elysia marginata
Elysia chlorotica
Mytilus edulis
Mytilus coruscus
Dreissena polymorpha
Lingula anatina
Octopus sinensis
Octopus bimaculoides
Littorina littorea
Branchiostoma belcheri
others
e)
Species Distribution [pest_rnd3_all_maker_transcripts_slimgo_nocon]
#BLAST Hits
0
10,000
20,000
30,000
40,000
50,000
60,000
70,000
80,000
Species
Pomacea canaliculata
Batillaria attramentaria
Haliotis rufescens
Biomphalaria glabrata
Haliotis rubra
Gigantopelta aegis
Patella vulgata
Aplysia californica
Mizuhopecten yessoensis
Crassostrea gigas
Crassostrea virginica
Bulinus truncatus
Pecten maximus
Mercenaria mercenaria
Mytilus galloprovincialis
Plakobranchus ocellatus
Ostrea edulis
Candidula unifasciata
Lottia gigantea
Elysia marginata
Elysia chlorotica
Mytilus edulis
Mytilus coruscus
Dreissena polymorpha
Lingula anatina
Octopus sinensis
Octopus bimaculoides
Littorina littorea
Branchiostoma belcheri
others
b)
Top-Hit Species Distribution [pest_rnd3_all_maker_transcripts_slimGO]
#BLAST Top-Hits
0
2,500
5,000
7,500
10,000
Species
Pomacea canaliculata
Batillaria attramentaria
Haliotis rufescens
Littorina littorea
Aplysia californica
Haliotis rubra
Gigantopelta aegis
Plakobranchus ocellatus
Elysia marginata
Patella vulgata
Biomphalaria glabrata
Lottia gigantea
Candidula unifasciata
Mercenaria mercenaria
Elysia chlorotica
Bulinus truncatus
Mytilus coruscus
Ostrea edulis
Crepidula fornicata
Mytilus galloprovincialis
Mytilus edulis
Crassostrea gigas
Sulfurovum sp.
Mizuhopecten yessoensis
Crassostrea virginica
Branchiostoma belcheri
Dreissena polymorpha
Pecten maximus
Escherichia coli
others
f)
Top-Hit Species Distribution [pest_rnd3_all_maker_transcripts_slimgo_nocon]
#BLAST Top-Hits
0
2,500
5,000
7,500
10,000
Species
Pomacea canaliculata
Batillaria attramentaria
Haliotis rufescens
Littorina littorea
Aplysia californica
Haliotis rubra
Gigantopelta aegis
Plakobranchus ocellatus
Elysia marginata
Patella vulgata
Biomphalaria glabrata
Lottia gigantea
Candidula unifasciata
Mercenaria mercenaria
Elysia chlorotica
Bulinus truncatus
Mytilus coruscus
Ostrea edulis
Crepidula fornicata
Mytilus galloprovincialis
Mytilus edulis
Crassostrea gigas
Sulfurovum sp.
Mizuhopecten yessoensis
Crassostrea virginica
Branchiostoma belcheri
Dreissena polymorpha
Pecten maximus
Escherichia coli
others
c)
Species Distribution [pkait_rnd3_all_maker_transcripts_slimGO]
#BLAST Hits
0
25,000
50,000
75,000
100,000
125,000
150,000
175,000
Species
Pomacea canaliculata
Batillaria attramentaria
Haliotis rufescens
Elysia marginata
Haliotis rubra
Gigantopelta aegis
Biomphalaria glabrata
Aplysia californica
Mizuhopecten yessoensis
Crassostrea gigas
Crassostrea virginica
Bulinus truncatus
Pecten maximus
Mytilus galloprovincialis
Plakobranchus ocellatus
Mercenaria mercenaria
Ostrea edulis
Candidula unifasciata
Lottia gigantea
Elysia chlorotica
Mytilus edulis
Mytilus coruscus
Patella vulgata
Dreissena polymorpha
Littorina littorea
Lingula anatina
Branchiostoma belcheri
Octopus bimaculoides
Octopus sinensis
others
g)
Species Distribution [pkait_rnd3_all_maker_transcripts_slimgo_nocon]
#BLAST Hits
0
25,000
50,000
75,000
100,000
125,000
150,000
175,000
Species
Pomacea canaliculata
Batillaria attramentaria
Haliotis rufescens
Elysia marginata
Haliotis rubra
Gigantopelta aegis
Biomphalaria glabrata
Aplysia californica
Mizuhopecten yessoensis
Crassostrea gigas
Crassostrea virginica
Bulinus truncatus
Pecten maximus
Mytilus galloprovincialis
Plakobranchus ocellatus
Mercenaria mercenaria
Ostrea edulis
Candidula unifasciata
Lottia gigantea
Elysia chlorotica
Mytilus edulis
Mytilus coruscus
Patella vulgata
Dreissena polymorpha
Littorina littorea
Lingula anatina
Branchiostoma belcheri
Octopus bimaculoides
Octopus sinensis
others
d)
Top-Hit Species Distribution [pkait_rnd3_all_maker_transcripts_slimGO]
#BLAST Top-Hits
0
2,500
5,000
7,500
10,000
12,500
15,000
17,500
Species
Pomacea canaliculata
Batillaria attramentaria
Elysia marginata
Littorina littorea
Apostichopus japonicus
Haliotis rufescens
Haliotis rubra
Gigantopelta aegis
Aplysia californica
Sulfurovum sp.
Plakobranchus ocellatus
Gammaproteobacteria bacterium
Patiria miniata
Biomphalaria glabrata
Elysia chlorotica
Lottia gigantea
Candidula unifasciata
Bulinus truncatus
Mercenaria mercenaria
Branchiostoma belcheri
Crassostrea gigas
Pecten maximus
Mytilus galloprovincialis
Patella vulgata
Mytilus edulis
Mizuhopecten yessoensis
Ostrea edulis
Dreissena polymorpha
Crassostrea virginica
others
h)
Top-Hit Species Distribution [pkait_rnd3_all_maker_transcripts_slimgo_nocon]
#BLAST Top-Hits
0
2,500
5,000
7,500
10,000
12,500
15,000
17,500
Species
Pomacea canaliculata
Batillaria attramentaria
Elysia marginata
Littorina littorea
Apostichopus japonicus
Haliotis rufescens
Haliotis rubra
Gigantopelta aegis
Aplysia californica
Sulfurovum sp.
Plakobranchus ocellatus
Gammaproteobacteria bacterium
Patiria miniata
Biomphalaria glabrata
Elysia chlorotica
Lottia gigantea
Candidula unifasciata
Bulinus truncatus
Mercenaria mercenaria
Branchiostoma belcheri
Crassostrea gigas
Pecten maximus
Mytilus galloprovincialis
Patella vulgata
Mytilus edulis
Mizuhopecten yessoensis
Ostrea edulis
Dreissena polymorpha
Crassostrea virginica
others

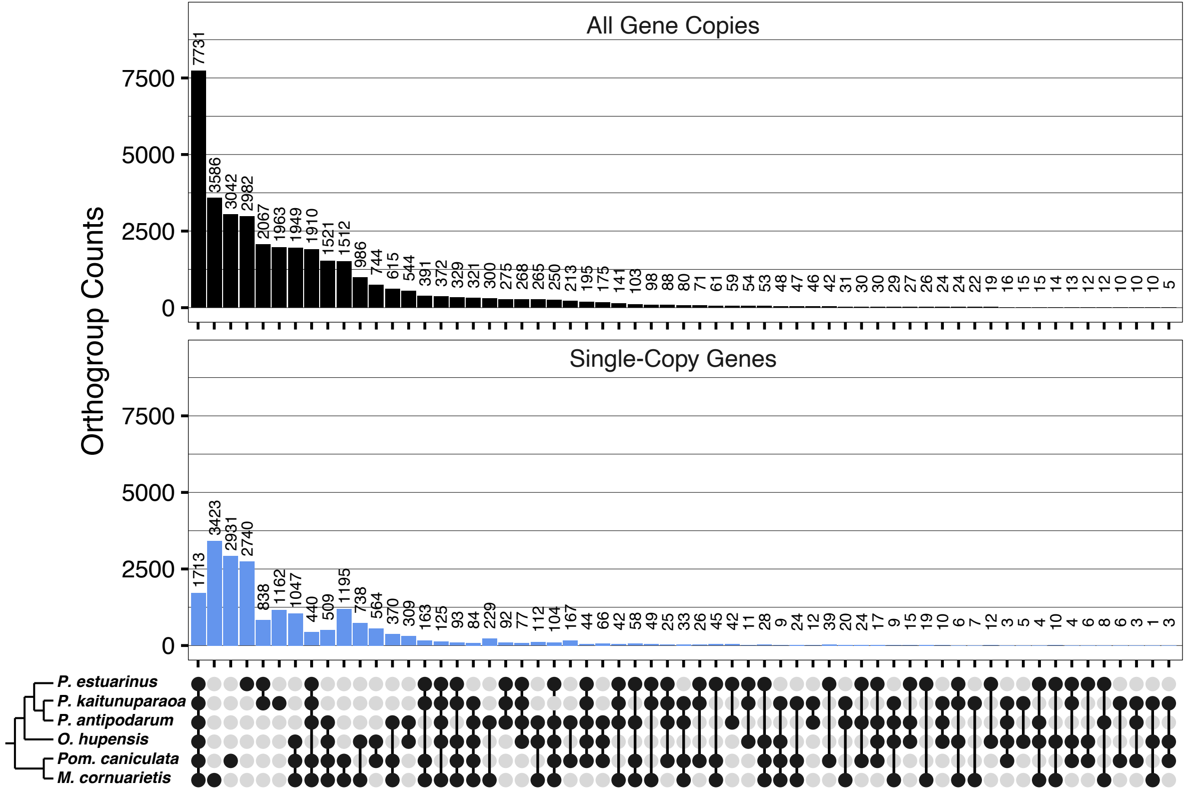

All Gene Copies
Single-Copy Genes
Orthogroup Counts
7500
5000
2500
0
7731 3586 3042 2982 2067 1963 1949 1910 1521 1512 986 744 615 544 391 372 329 321 300 275 268 265 250 213 195 175 141 103 98 88 80 71 61 59 54 53 48 47 46 42 31 30 30 29 27 26 24 24 22 19 16 15 15 14 13 12 12 10 10 10 5
1713 3423 2931 2740 838 1162 1047 440 509 1195 738 564 370 309 163 125 93 84 229 92 77 112 104 167 44 66 42 58 49 25 33 26 45 42 11 28 9 24 12 39 20 24 17 9 15 19 10 6 7 12 3 5 4 10 4 6 8 6 3 1 3
P. estuarinus
P. kaitunuparaoa
P. antipodarum
O. hupensis
Pom. caniculata
M. cornuarietis

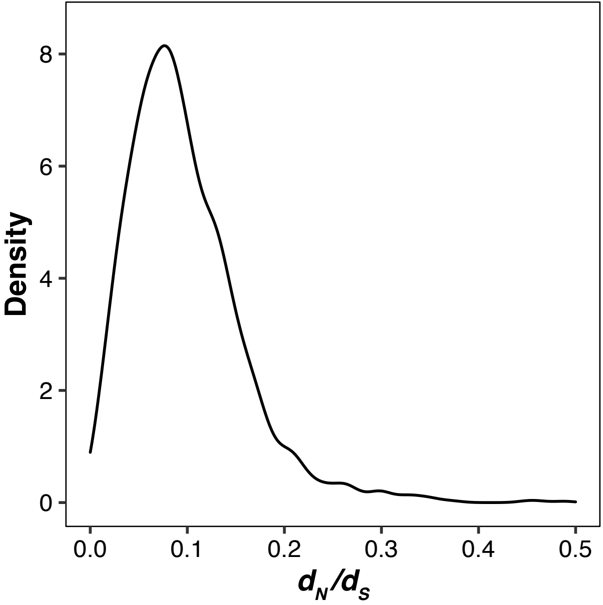

Density
8
6
4
2
0
0.0
0.1
0.2
0.3
0.4
0.5
d_N/d_S

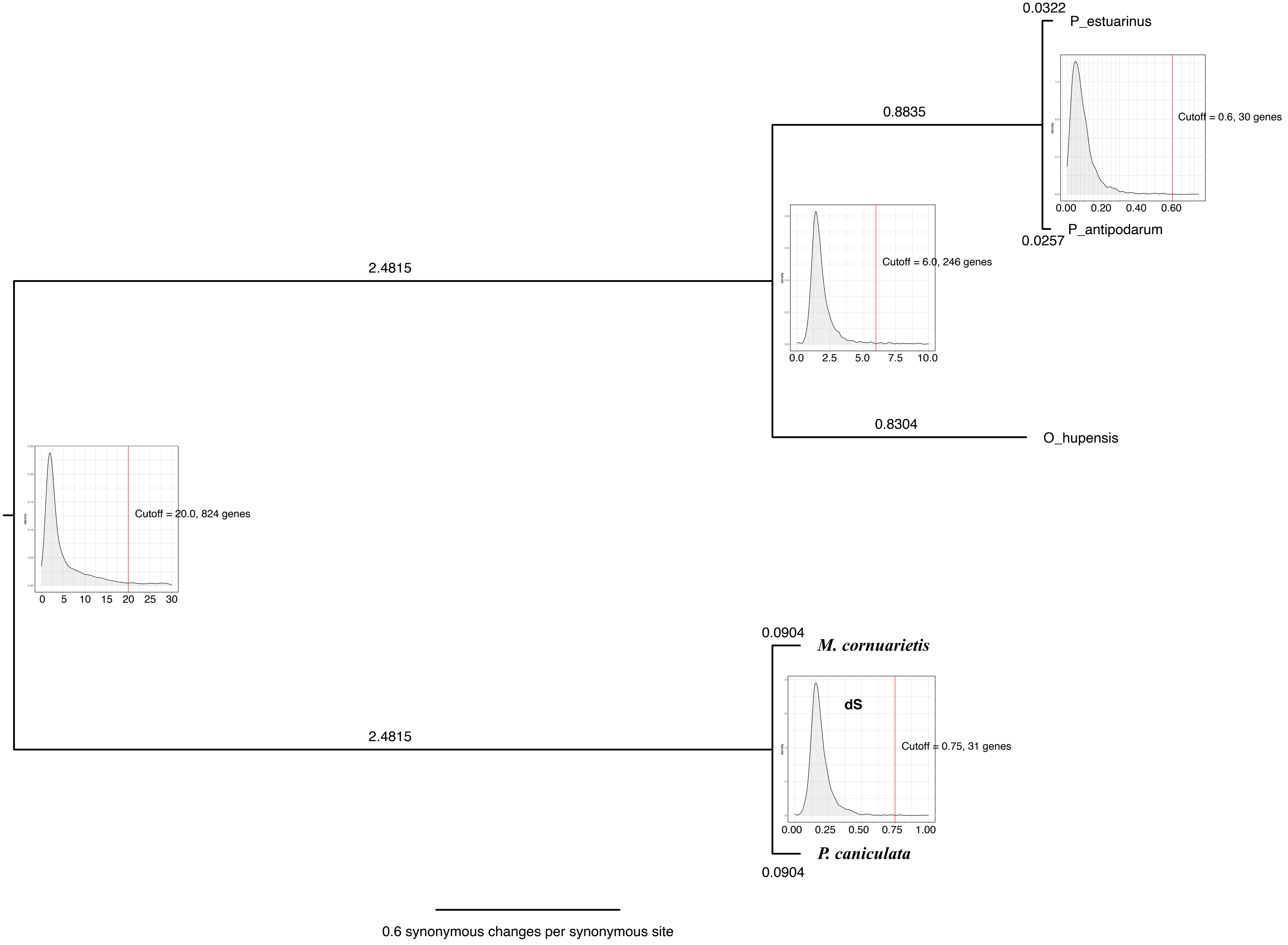
0.0322
P_estuarinus
0.8835
Cutoff = 0.6, 30 genes
0.00 0.20 0.40 0.60
P_antipodarum
0.0257
2.4815
Cutoff = 6.0, 246 genes
0.0 2.5 5.0 7.5 10.0
0.8304
O_hupensis
Cutoff = 20.0, 824 genes
0 5 10 15 20 25 30
0.0904
M. cornuarietis
dS
2.4815
Cutoff = 0.75, 31 genes
0.00 0.25 0.50 0.75 1.00
P. caniculata
0.0904
0.6 synonymous changes per synonymous site

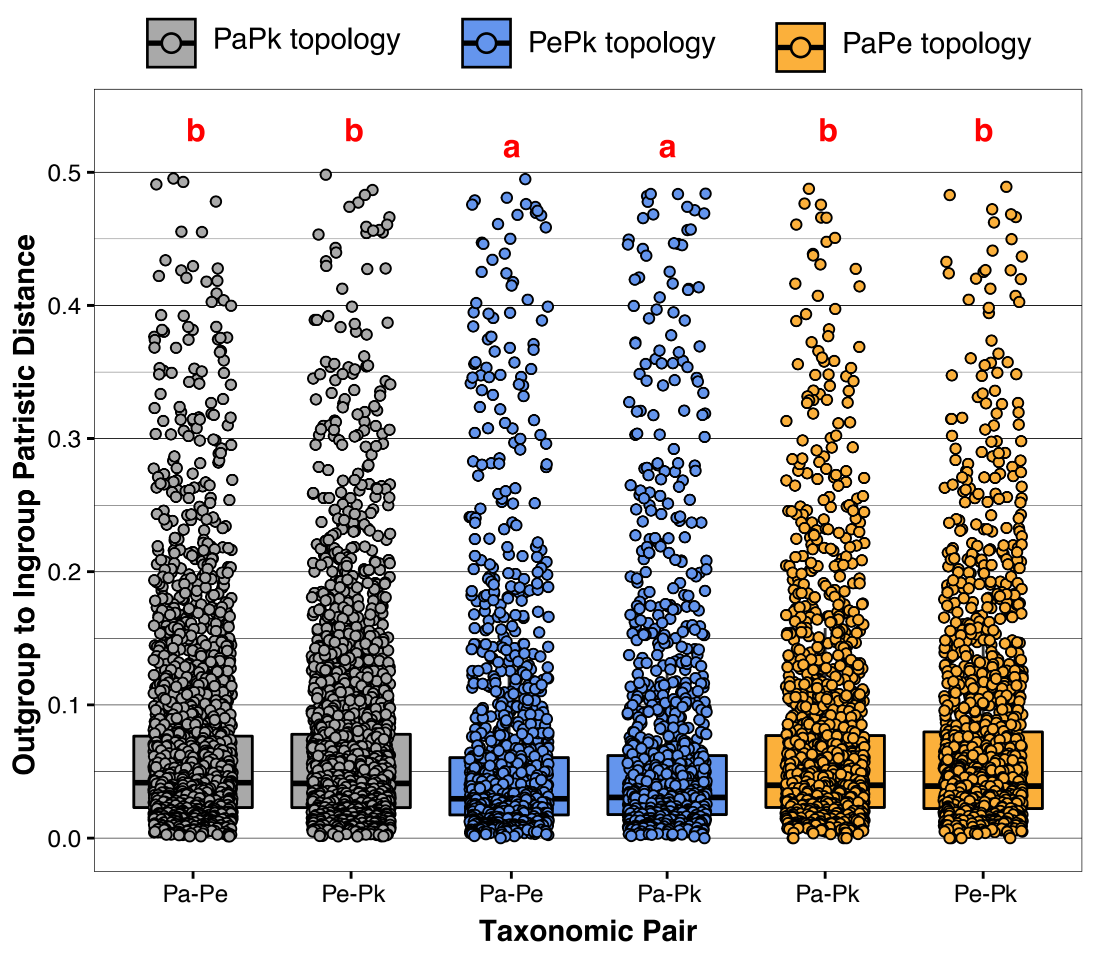
PaPk topology
PePk topology
PaPe topology
Outgroup to Ingroup Patristic Distance
0.0
0.1
0.2
0.3
0.4
0.5
b
b
a
a
b
b
Pa-Pe
Pe-Pk
Pa-Pe
Pa-Pk
Pa-Pk
Pe-Pk
Taxonomic Pair

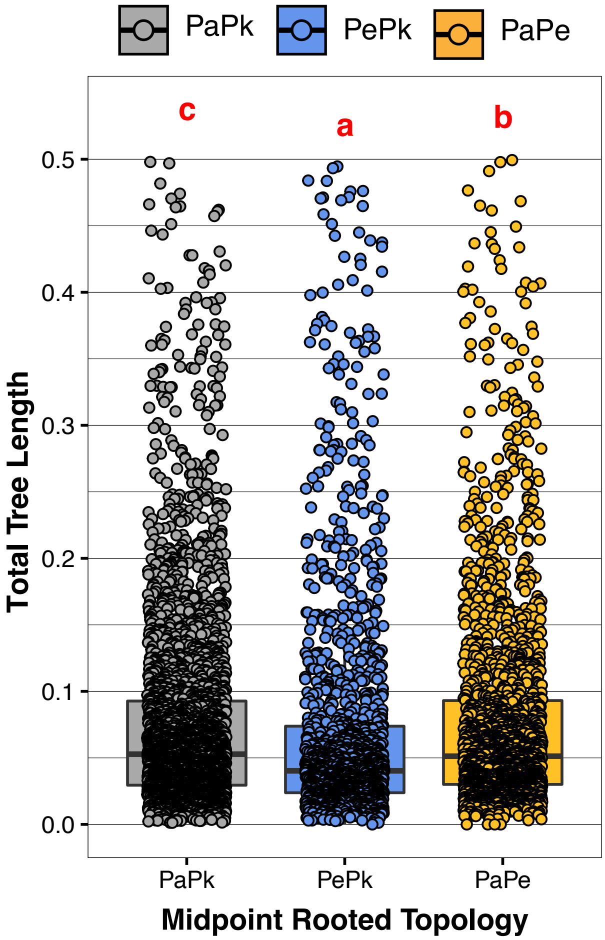

PaPk
PePk
PaPe
c
a
b
0.5
0.4
0.3
0.2
0.1
0.0
Total Tree Length
PaPk
PePk
PaPe
Midpoint Rooted Topology

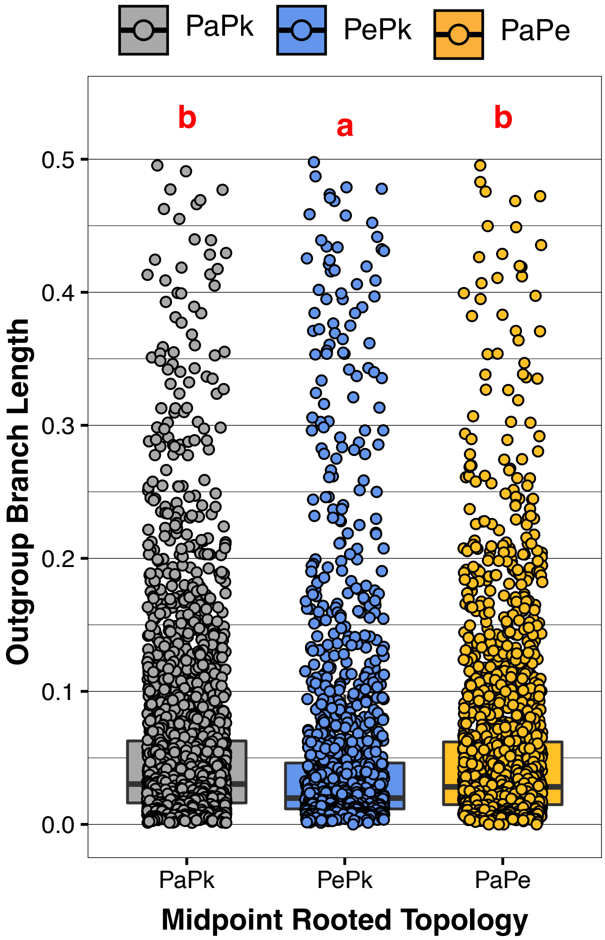
PaPk
PePk
PaPe
b
a
b
0.5
0.4
0.3
0.2
0.1
0.0
Outgroup Branch Length
PaPk
PePk
PaPe
Midpoint Rooted Topology

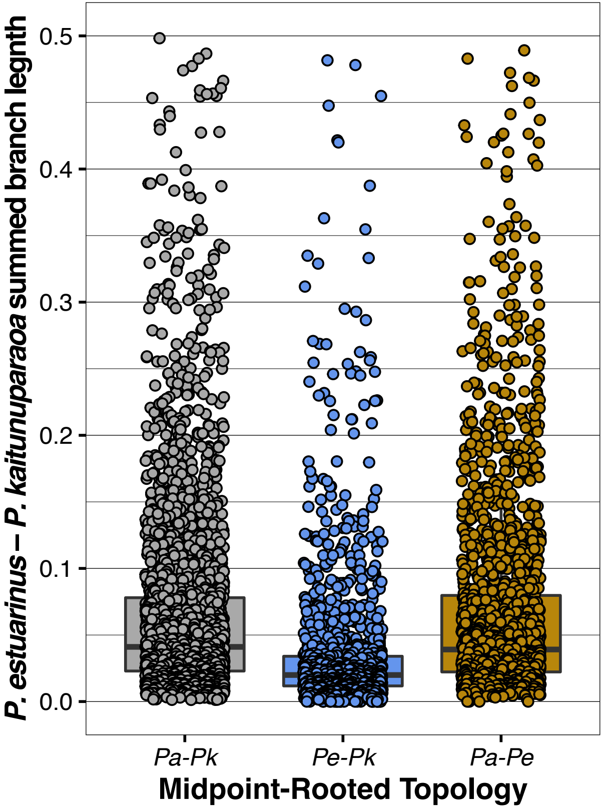

P. estuarinus – P. kaitunuparaoa summed branch legnth
0.5
0.4
0.3
0.2
0.1
0.0
Pa-Pk
Pe-Pk
Pa-Pe
Midpoint-Rooted Topology
